# The Link between Autoimmunity and Lymphoma: Does NOTCH Signaling Play a Contributing Role?

**DOI:** 10.3389/fonc.2015.00051

**Published:** 2015-02-24

**Authors:** Christina Arieta Kuksin, Lisa M. Minter

**Affiliations:** ^1^Department of Veterinary and Animal Sciences, University of Massachusetts Amherst, Amherst, MA, USA; ^2^Program in Molecular and Cellular Biology, University of Massachusetts Amherst, Amherst, MA, USA

**Keywords:** NOTCH, NF-κB, autoimmunity, lymphoma

## Abstract

An association between certain autoimmune conditions and increased risk of developing lymphoma is well documented. Recent evidence points to NOTCH signaling as a strong driver of autoimmunity. Furthermore, a role for NOTCH in various lymphomas, including classical Hodgkin lymphoma, non-Hodgkin lymphoma, and T cell lymphoma has also been described. In this mini-review, we will outline what is known about involvement of NOTCH signaling in those autoimmune conditions, such as rheumatoid arthritis and primary Sjörgren’s syndrome, which show an increased risk for subsequent diagnosis of lymphoma. Furthermore, we will detail what is known about the lymphomas associated with these autoimmune conditions and how aberrant or sustained NOTCH signaling in the immune cells that mediate these diseases may contribute to lymphoma.

It has long been recognized that patients with particular autoimmune disorders and inflammatory conditions have an increased risk for developing specific lymphomas. Generally, the link between autoimmune diseases and developing lymphoma is correlative; however, there exists a subset of autoimmune diseases for which the risk of subsequent lymphoma development is strong (1, 2). This review will focus on four autoimmune disorders that have a well-established association with lymphoma occurrence: rheumatoid arthritis (RA), systemic lupus erythematosus (SLE), primary Sjörgren’s syndrome (pSS), and celiac disease. We will focus on the involvement of the NOTCH pathway in these disorders, as well as the developing lymphomas, and postulate how NOTCH signaling may be one of the pre-disposing factors in their development.

The NOTCH family comprises four transmembrane receptors (NOTCH1-4), which orchestrate multiple cell fate decisions. NOTCH receptors engage one of five ligands, Jagged (Jag) 1, 2 or Delta-like (DLL) 1, 3, 4, and are ultimately cleaved by γ-secretase, liberating the signaling-competent, intracellular domain from the membrane (NOTCH^IC^). NOTCH^IC^ may then interact with proteins in the cytoplasm, such as NF-κB, or translocate to the nucleus to mediate downstream gene transcription, including the classical targets, Hairy enhancer of split (Hes), and Hes-related with YRPW motif [Hey; (3–5)]. NOTCH has been shown to be important in hematopoietic lineage decisions. During lymphocyte ontogeny, NOTCH1 promotes adoption of a T cell fate at the expense of B cell development (6). Dysregulated NOTCH signaling is implicated in many diseases, including autoimmune disorders and various malignancies (7, 8).

Rheumatoid arthritis is a chronic inflammatory condition characterized by painful swelling of affected joints. It has been most strongly associated with subsequent development of diffuse large B cell lymphoma (DLBCL), a specific type of non-Hodgkin’s lymphoma [NHL; (1)]. Extensive evidence indicates NOTCH signaling plays an important role in the pathogenesis of RA. Compared to healthy joints, all four NOTCH homologs show aberrant expression in the inflamed synovium of RA patients (9, 10). In particular, NOTCH1 and NOTCH3 are highly expressed, as are the ligands DLL1 and Jag1, on the synovial lining and sublining of synovial hyperplastic lesions. NOTCH1 and NOTCH3 expression overlapped with Jag1 expression (11). Moreover, in the lymphoid follicles of these patients, NOTCH1 was detected in T and B cells. Multiple studies have reported activated, cleaved NOTCH1^IC^ in the synovium and in autoreactive T cells (10–12). Additionally, NOTCH1^IC^ and NOTCH4^IC^ have been found in RA synoviocytes (13).

Inhibiting NOTCH signaling ameliorates pro-inflammatory responses in RA. Blocking NOTCH cleavage using γ-secretase inhibitors (GSI) reduces Th1- and Th17-mediated inflammatory responses *in vitro*, as well as in a mouse model of RA (14). GSI or NOTCH3-neutralizing antibodies can reduce T cell proliferation and pro-inflammatory cytokine production (15). GSI also reduces TNF-induced IL-6 production and cell proliferation in RA synoviocytes (12, 16). These data provide evidence NOTCH signaling is important for RA progression by regulating cytokine production and cell proliferation.

The mechanism(s) by which NOTCH signaling is sustained or how it acts to facilitate RA progression remains an area of active investigation. Samples from RA patients incubated with TNF upregulated NOTCH and ligand expression (12, 13, 16). Interestingly, individual NOTCH ligands have differential effects on proliferation and cytokine production by RA cells. Culturing cells with DLL1 promotes T cell proliferation and pro-inflammatory cytokine production in RA (15, 16). Conversely, administering soluble Jag1 provides a negative signal to CD8^+^ T cells and reduces disease symptoms in a mouse model of RA (17). NOTCH may also be acting to regulate NF-κB activation in synoviocytes. Nuclear NOTCH1 has been shown to form a complex with RBP-Jκ and reverse NF-κB2 promoter suppression (18). Collectively, these date provide clear evidence NOTCH signaling is important for the pathogenesis of RA.

Although not as tightly linked as for patients with RA, patients with SLE also display an increased risk of developing DLBCL and classical Hodgkin’s lymphoma [cHL; (1)]. Autoimmune SLE is characterized by the production of autoantibodies and immune-complex deposition that affect multiple organs. Increased numbers of macrophages, termed “M2b” that are defined by a signature profile of high IL-10/TNF/IL-1β/IL-6/MCP-1 production and low IL-12 secretion have been described in SLE (19). M2b macrophages also express high levels of NOTCH1^IC^ as well as elevated *Hes1* and *Hey1*. Furthermore, GSI treatment in a mouse model of SLE impaired macrophage differentiation into the M2b phenotype and ameliorated lupus-associated symptoms, which included reduced anti-dsDNA titers, decreased kidney scores, and attenuated IgG deposition (19). It is not exactly known how NOTCH signaling may be driving this pro-inflammatory response, but macrophages stimulated by activated, lymphocyte-derived self-apoptotic DNA (ALD-DNA) enhanced NOTCH1 signaling and was accompanied by increased nuclear translocation of NF-κB p50. In contrast, GSI treatment strongly downregulated NF-κB activity, supporting the notion that NOTCH1 is driving NF-κB associated pro-inflammatory responses in M2b macrophages (19).

In addition to its implication in M2b macrophage differentiation, NOTCH signaling may also play an important role in autoreactive lymphocytes associated with SLE. GSI treatment decreased CD4^+^ T cell proliferation in the lymph nodes and spleen, and lowered cytokine secretion and monocyte chemoattractant protein-1 (MCP-1) production (20). The authors of this report speculated that inhibiting NOTCH signaling altered disease-associated double negative T cells which, in turn, ameliorated disease symptoms. GSI treatment also decreased MCP-1 levels, which have been shown to contribute significantly to the Th1 response that drives the autoimmune manifestations in murine SLE (21–23). In human SLE, NOTCH1 expression is downregulated in T cells from patients with active SLE (24, 25). Decreased NOTCH1 mRNA and protein expression was determined to be due to epigenetic modification of the *NOTCH1* promoter, including histone and CpG DNA methylation and transcriptional repression mediated by CREMα (24). Reduced NOTCH1 expression was associated with decreased proliferation and lower CD25 and Foxp3 expression following *in vitro* stimulation (25). NOTCH1 is important for generating *de novo* regulatory T cells (26), and it is intriguing to speculate that impaired NOTCH1 signaling may adversely affect regulatory T cells in SLE, either in number or function. Abrogated NOTCH1 signaling was also accompanied by an observed increase in IL-17A expression in SLE patients (24). It is therefore possible that NOTCH signaling drives SLE pathogenesis by inhibiting regulatory T cell development, thus allowing the generation of autoreactive, IL-17A-producing T cells.

Patients with pSS experience chronic autoimmune destruction of the exocrine glands, specifically the salivary and lacrimal glands. Cases of pSS are associated with an increased risk of DLBCL and marginal zone lymphoma, specifically, MALT lymphoma of the parotid gland (1). Not much is known about the role of NOTCH in pSS, although *NOTCH2* mRNA has been detected in marginal zone B cells in the salivary gland and in tonsil germinal centers in pSS patients (27). Clusters of transitional type II B cells in salivary glands expressed *NOTCH2* and *BLIMP1* mRNA and these cells behaved like marginal zone B cells (28). Although evidence defining a contribution for NOTCH signaling in the pathogenesis Sjörgren’s syndrome is not abundant, NOTCH signaling is important in the development of salivary glands, a process, which is defective in pSS (29). Furthermore, abnormalities in NF-κB signaling have been observed, with polymorphisms in genes associated with the NF-κB pathways also documented in pSS patients (30–32). Given that NF-κB and NOTCH pathways are known to interact, it is possible that dysregulated NF-κB signaling may lead to dysregulated NOTCH signaling. Additional investigation of autoimmune mechanisms that potentiate pSS will be useful to further define any involvement of aberrant NOTCH signaling in this disease.

As with pSS, little is known about NOTCH signaling in celiac disease, which results from chronic immune stimulation in response to dietary proteins in patients with a genetic predisposition. Celiac disease is a distinctly T cell-mediated condition and the TCRs responsible for recognizing and responding to the inciting antigen, gliadin, a component of the gluten protein, have been recently identified (33). Celiac disease is also associated with development of non-Hodgkin’s T cell lymphoma. Intestinal biopsies from patients with active celiac disease, including those on a gluten free diet, revealed decreased *NOTCH1* and *HES1* mRNA expression in goblet cells, compared to healthy patients (34). Furthermore, dysregulated NOTCH activity is related to the immune-mediated pathogenesis of irritable bowel diseases, such as ulcerative colitis and Crohn’s disease (35). Polymorphisms in the NF-κB-related genes, *REL* and *OLIG3/TNFAIP3* have been identified and were shown to contribute to celiac disease (36). Should NOTCH signaling in gut T cells prove to be pathogenic in celiac disease, a significant treatment challenge will likely result: that is how to balance decreasing aberrant NOTCH signaling in autoreactive T cells with maintaining a healthy level of NOTCH signaling in intestinal goblet cells (37).

A diagnosis of any of the four autoimmune diseases described above leaves patients at increased risk of developing lymphoma (Figure 1). It is interesting to note, however, that aggressive lymphocyte neoplasms are associated with these autoimmune diseases, rather than more the indolent chronic lymphocytic leukemias (1). DLBCL is the most common type of NHL. This fast-growing lymphoma is characterized by large B cells that grow diffusely throughout the lymph nodes. While NOTCH signaling has a clear role in T cell proliferation and T cell malignancies, dysregulated NOTCH signaling in B cell lymphoma is more controversial (38). The TMD8 cell line was developed from a patient with DLBCL. These cells express NOTCH1, NOTCH2, Jagged1, DLL4, and constitutively express *HES1* mRNA. Most DLBCLs express Epstein–Barr proteins (EBNA2), which mimic NOTCH signaling to promote HES expression. However, TMD8 cells do not express EBNA2; therefore, the constitutive *HES1* expression is thought to be driven by NOTCH signaling. Treating this cell line with GSI impaired cell growth and reduced expression of *HES1*. However, one caveat of this study is that GSI did not fully inhibit NOTCH1 cleavage, which might indicate a slow rate of NOTCH1 protein degradation. Alternatively, the biological effects seen may result from GSI acting on another target of γ-secretase, such as NOTCH2; however, NOTCH2 cleavage after GSI treatment was not assessed (39). Although NOTCH1^IC^ could be detected in the TMD8 cell line, a tissue microarray containing 68 well-characterized DLBCLs revealed none that stained positively for NOTCH1^IC^ (40). This may suggest that family members other than NOTCH1 are dysregulated in these tumors, but more studies are needed to ascertain the degree to which activated NOTCH drives DLBCL.

**Figure 1:**
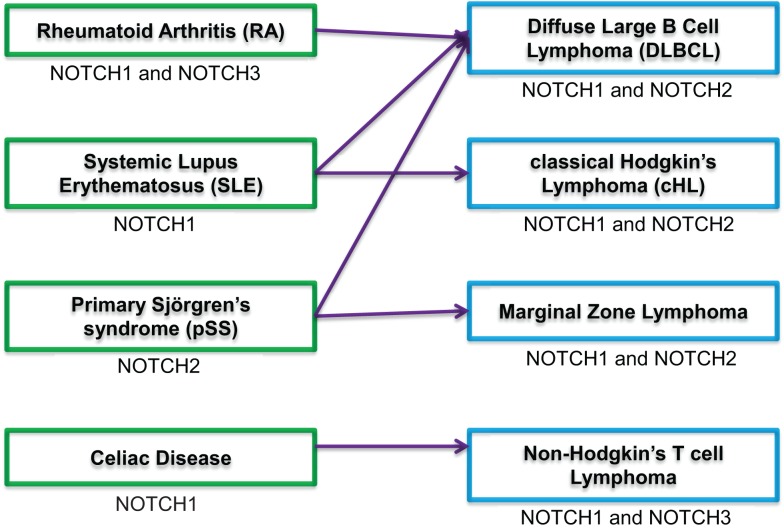
**Potential association of autoimmune conditions and lymphoma**. Schematic showing four autoimmune diseases (green boxes, left side) associated with increased risk for developing lymphomas [blue boxes, right side; (1, 2)]. The arrows indicate correlation between specific autoimmune diseases and most frequently associated lymphoma. NOTCH signaling component(s) dysregulated in autoimmune conditions or lymphoma is indicated under each box.

In studies of patients with DLBCL, mutations in the heterodimerization and PEST domains of *NOTCH2* were found in a small (8%) percentage. Of this cohort, 40% had increased copy numbers of the *NOTCH2* allele carrying the PEST domain mutation, and in one patient the total copy number of *NOTCH2* was increased. Furthermore, mutant NOTCH2 receptors showed increased activity when stimulated with NOTCH ligands *in vitro* (41). A SNP variation in 13q12, which contains the NOTCH signaling mediator, *LNX2*, has also been identified. This gene encodes a PDZ domain-containing zinc finger 1 protein, which may function as an E3 ubiquitin ligase. Elevated levels of LNX family members were shown to promote ubiquitin–proteasomal degradation of the NOTCH negative regulator, NUMB, resulting in enhanced NOTCH signaling (42). NF-κB expression and activation is also high in DLBCL tumors (43–46). Thus, both NOTCH and NF-κB may play a role in DLBCL progression.

Classical Hodgkin’s lymphoma is characterized by the presence of very large cells called Hodgkin and Reed-Sternberg (HRS) cells, although other abnormal cell types may also be present. NOTCH signaling has been shown to play a prominent role in cHL pathogenesis. NOTCH1 and NOTCH2 are aberrantly expressed in HRS cells and are stimulated through Jagged1 to promote proliferation and protect from apoptosis (47). HRS cells are known to express Jagged1, Jagged2, DLL1, DLL3, and DLL4 (47, 48). Finally, an absence of the NOTCH1 inhibitor DELTEX1, together with increased expression of the NOTCH co-activator, MAML, likely drive NOTCH signaling in HRS cells (48, 49).

NOTCH and NF-κB signaling pathways have been shown to cooperate, resulting in cHL pathogenesis. HRS cells are characterized by high levels of nuclear NF-κB p50/p65 dimers, which are required for proliferation, protection from apoptosis, and growth in lymphoma mouse models (50–52). In addition, mutations in NF-κB regulators, such as IκBα, IκBε, or IκB kinase (IKK)α, contribute to constitutive activation of canonical NF-κB signaling in HRS cells (51, 53, 54). Inhibiting NOTCH activity in HRS cells using GSI induces apoptosis through simultaneous targeting of the NOTCH and alternative NF-κB signaling pathways (55). Thus, NOTCH signaling conspires to drive cHL pathology and inhibiting NOTCH signaling may have positive implications for treatment.

MALT lymphoma of the parotid gland represents a small subset of head and neck NHL. A diagnosis of Sjörgren’s syndrome is associated with a 1000-fold increase in the risk of parotid gland marginal zone lymphoma (56). Although no studies have explored how NOTCH signaling may contribute to development of MALT lymphoma of the parotid gland, there have been studies examining MALT lymphoma in the spleen (SMZL). In 30% of SMZL cases, alterations in genes of the NOTCH pathway emerged as highly recurrent. *NOTCH2*, a key regulator of marginal zone B cell development, was identified as the most frequently mutated gene, occurring in approximately 20% of cases, often resulting in disruption of the PEST domain (57–59). In addition to NOTCH2, genetic lesions in other modulators or members of the NOTCH pathway were also observed in SMZL, including *SPEN*, *DELTEX1*, and *NOTCH1* (58). Mutations in members of the NF-κB pathway have also been implicated in MALT lymphoma (60–62). In fact, somatic mutations of TNFAIP3 (A20) protein, which plays a key role in controlling NF-κB activation, have been observed in the MALT lymphoma subtype frequently associated with SS (60). More studies are needed to ascertain whether and how NOTCH signaling drives MALT lymphomas associated with pSS.

The majority of systemic autoimmune diseases predispose to developing lymphomas of B cell origin. However, patients with autoimmune diseases of the gastrointestinal tract, such as celiac disease, exhibit a higher risk of NHL T cell lymphoma (63), a subset of NHL arising from a single T cell clone. Mutations in *NOTCH1* have been found with high frequency in all major oncogenic subclasses of human T cell lymphomas, suggesting NOTCH1 plays a significant role in lymphomagenesis (64). In a small study, half of the T cell lymphomas examined carried activating *NOTCH1* mutations and/or mutations in the NOTCH-inhibiting *FBXW7*, which encodes an ubiquitin ligase that degrades NOTCH (65, 66). In addition, *NOTCH3* mRNA has also been noted in patients with T cell lymphoma, and thymocyte-specific *NOTCH3^IC^* transgenic mice develop T cell lymphoma of the spleen and lymph nodes (67, 68). Interestingly, *NOTCH2* mRNA has been shown to be decreased in γ-irradiation-induced T cell lymphomas (69). T cell lymphomas treated *in vitro* with GSI showed growth inhibition and caspase-mediated apoptosis (65, 70). A role for NOTCH–NF-κB interaction has been described in mouse models of T cell lymphoma (67), and elevated NF-κB activity in the primary cells of patients with T cell lymphoma has been described in cases of acute but not chronic disease (71). As with other types of lymphomas discussed here, NOTCH signaling appears to play an important role in T cell survival in lymphomas (47).

Patients with certain autoimmune diseases face an increased risk of developing aggressive lymphomas. Based on the evidence presented here, we suggest that dysregulated NOTCH signaling is implicated both in the autoimmune responses and the associated lymphomas. Chronic antigenic stimulation and inflammation define the immunological environment of autoimmunity and are factors that can precipitate the onset of lymphoma. Furthermore, convergence of the NOTCH and NF-κB signaling pathways likely promote both the pro-inflammatory environment and transformation of stimulated clones. Inhibiting the NOTCH pathway in autoimmune disease may act to attenuate both conditions since NOTCH signaling may be a mediator both of the autoimmune process and the lymphomas that arise subsequently.

## Conflict of Interest Statement

The authors declare that the research was conducted in the absence of any commercial or financial relationships that could be construed as a potential conflict of interest.
